# 2q37 deletion syndrome in a Colombian patient with macrocephaly: a case report

**DOI:** 10.1186/s12887-022-03620-8

**Published:** 2022-10-04

**Authors:** Sebastian Giraldo-Ocampo, Harry Pachajoa

**Affiliations:** 1 Universidad del Valle, Cali, Colombia; 2 Genetics Division, Fundación Valle del Lili, Cali, Colombia; 3 Centro de Investigaciones en Anomalías Congénitas Y Enfermedades Raras (CIACER), Universidad Icesi, Calle 18 No. 122-135 Pance, Cali, Colombia; 4 Centro de Investigaciones Clínicas, Fundación Valle del Lili, Cali, Colombia

**Keywords:** Albright hereditary osteodystrophy-like syndrome (AHO-like), Brachydactyly-mental retardation syndrome (BDMR), Macrocephaly, Case report

## Abstract

**Background:**

2q37 deletion syndrome is a rare autosomal dominant disorder caused by deletions in the 2q37 cytobands leading to developmental delay, intellectual disability, behavioral abnormalities and dysmorphic craniofacial features with more than 115 patients described worldwide.

**Case presentation:**

We describe a Colombian 3-year-old patient with verbal communication delay, umbilical hernia, facial dysmorphic features, hypotonia, and macrocephaly with normal magnetic resonance imaging. Microarray-based comparative genomic hybridization revealed a 5.9 Mb deletion in the 2q37.2 and 2q37.3 regions, eliminating 60 protein-coding genes in one of her chromosomes 2 and allowing the diagnosis of 2q37 deletion syndrome in this patient. Therapeutic interventions so far were the surgical correction of the umbilical hernia.

**Conclusions:**

Genetic tests are important tools for the diagnosis of clinically complex and infrequent conditions but also for timely diagnosis that allows appropriate surveillance, interventions, and genetic counseling. This case also provides information for expanding the phenotypical and genetic characterization of 2q37 deletion syndrome.

## Introduction

2q37 deletion syndrome (del2q37, OMIM: 600430), alternatively known as Albright hereditary osteodystrophy-like syndrome (AHO-like) or brachydactyly-mental retardation syndrome (BDMR), is a rare autosomal dominant genetic disorder first reported in 1995 [1] and since then, more than 115 cases worldwide have been published [2]. It is characterized by developmental delay, mild to moderate intellectual disability, behavioral abnormalities and dysmorphic craniofacial features [3]. Additional clinical manifestations include brachydactyly type E in about half of the patients, short stature, obesity, seizures, hypotonia, structural abnormalities of the central nervous system, the heart, trachea and gastrointestinal/genitourinary tract in almost one-third of affected individuals [3, 4].

Although deletions reported in most patients affect many genes, it seems that the loss of the Histone Deacetylase 4 (HDAC4) gene leads to haploinsufficiency and drives the phenotype typically seen in these patients (brachydactyly type E, developmental delays, and Behavioral Problems) [5, 6]. HDAC4 protein is involved in a wide variety of functions including gene transcription regulation, cell growth, survival and proliferation, chondrogenesis, osteoblast differentiation, muscle development, neuronal survival, pancreatic beta/delta-cell lineage specification and so on [7]. Given the low number of patients described in the literature up to date, more data describing the genotype–phenotype correlation of this syndrome is needed as well as the report of unusual symptoms that these patients may present. Here we report one of the few Colombian patients diagnosed with a heterozygous deletion of 5.9 Mb in the subtelomeric region in the long arm of chromosome 2 leading to 2q37 deletion syndrome with a unique clinical manifestation: macrocephaly.

## Case presentation

A 3-year-old female patient was born to nonconsanguineous parents, a healthy mother and a father with macrocephaly, by cesarean section without complication. Birth weight was 4500 gr (> 97th percentile) and birth length was 62 cm (> 97th percentile). Prenatal echography was indicative of macrosomia. In the past, the mother had four pregnancies with two different partners. In the first one, a healthy male was born (half-brother of the patient) and the second pregnancy was interrupted due to a large cystic hygroma in the fetal cranial base. The other two pregnancies were with the father of the patient but one of them was a miscarriage in the third semester with no clear cause. The patient did not crawl, presented sitting posture at four months and walked at 18 months of age.

At 3 years old, physical examination revealed normal psychomotor development for the patient’s age but presented a delay in verbal communication. The only behavioral abnormality referred to by the mother was the rising of arms in response to stimuli of joy and exaltation. Additional features noticed were macrocephaly (head circumference of 53.5 cm, > 99th percentile); an umbilical hernia, which was surgically corrected at 2 years of age; a defect of less than 1 cm in the abdominal wall; hypotonia and several dysmorphic features such as prominent forehead and occiput, thin and upturned eyebrows, posteriorly rotated ears, flat nasal bridge, low hanging columella, thin upper lip, downturned corners of mouth and breast hypertelorism. Hands and feet were normal. Weight was 18.5 kg (percentile 73) and length was 106 cm (percentile 81). Magnetic Resonance Imaging (MRI) of the brain, electromyography (EMG), echocardiogram and abdominal ultrasound were normal.

**Fig. 1:**
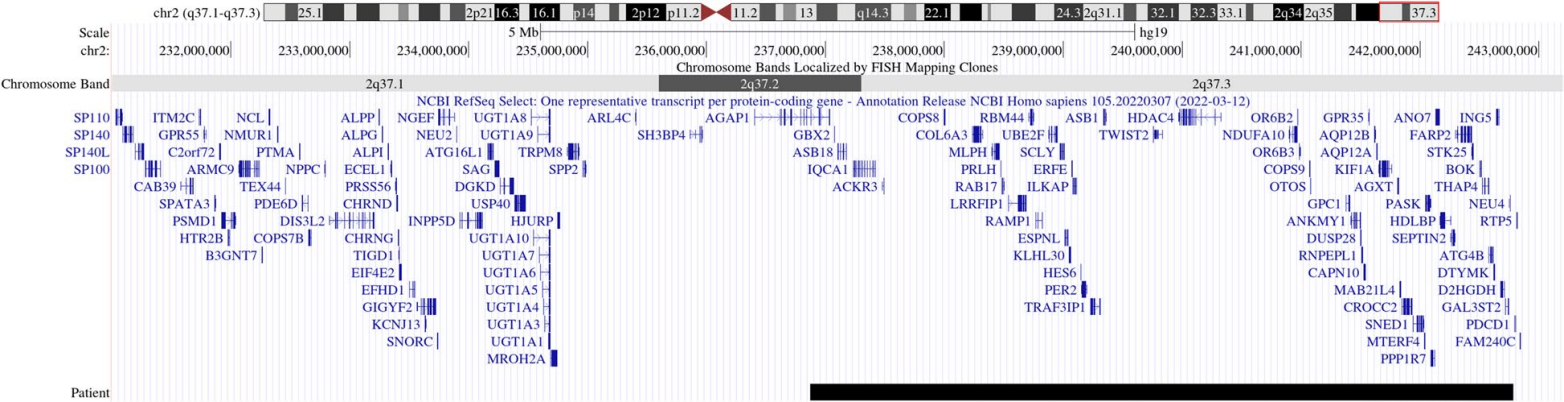
Map of the 2q37 deletion in the patient. Genes from chromosome bands 2q37.1 to 2q37.3 are shown. The map was create using UCSC Genome Browser on Human (GRCh37/hg19). Black bar indicates the length of the deletion found

Conventional chromosome analysis (karyotype) revealed 46, XX and therefore a Microarray-based comparative genomic hybridization (array-CGH) was performed. A 5.9 Mb deletion was found in the distal or subtelomeric region of the long arm of one of the chromosomes 2, comprising the genomic position 236869919 to 242782258, located in the 2q37.2 and 2q37.3 sub-bands (Fig. 1), allowing the diagnosis of chromosome 2q37 deletion syndrome (OMIM: 600430). Nearly 60 coding-protein genes are annotated in the National Center for Biotechnology Information (NCBI) database in the deleted region found in the patient. However, only 48 genes are annotated in the OMIM database, from which, 11 genes are associated with different pathologies (Table 1). Currently, the patient attends kindergarten and the mother reported a major improvement in verbal communication as she grows.

**Table 1:** Genetic alteration found in the patient and the genes deleted

ISCN 2016 formula	Type	Zygosity	Size (Mb)	OMIM genes in the region	CNV classification
arr[GRCh37] 2q37.2q37.3(236869919_ 242782258) × 1	Loss	heterozygous	5.912	AGAP1, GBX2, **ACKR3**, COPS8, **COL6A3**, **MLPH**, PRLH, RAB17, LRRFIP1, RAMP1, UBE2F, SCLY, ERFE, ILKAP, HES6, **PER2**, **TRAF3IP1**, ASB1, **TWIST2**, HDAC4, **NDUFA10**, COPS9, OTOS, GPC1, MIR149, RNPEPL1, **CAPN10**, GPR35, AQP12A, **KIF1A**, **AGXT**, SNED1, MTERF4, PASK, PPP1R7, ANO7, HDLBP, SEPTIN2, FARP2, STK25, BOK, THAP4, ATG4B, DTYMK, ING5, **D2HGDH**, GAL3ST2, NEU4	Pathogenic

Genes written in bold and underline text are associated with different disorders in the OMIM database

## Discussion and conclusions

We report a Colombian patient with a 5.9 Mb deletion in the 2q37 cytoband leading to 2q37 deletion syndrome. Del2q37 patients can present with a variable set of clinical manifestations including major malformations (congenital heart and skeletal malformations, gastrointestinal and renal anomalies and genitourinary and central nervous malformations) in nearly 30% of patients; neurodevelopmental and behavioral anomalies like hypotonia, seizure, epilepsy and autistic features that are frequently reported; typical facial dysmorphisms are reported in most cases and comprises prominent forehead, depressed nasal bridge, thin upper lip and arched eyebrows and late-onset abnormalities such as recurrent otitis and lower respiratory tract infection, umbilical and inguinal hernias, articulation dislocation, sparse or thin hair, joint laxity and eczema [8]. Our 3-year-old patient had the usual facial dysmorphism commonly reported, an umbilical hernia and a delay in verbal communication. The latter, as reported by the mother, is improving considerably as the patient grows. Aside from these manifestations, our patient presented with macrosomia at birth and currently presents macrocephaly with normal MRI, conditions not usually described as part of 2q37 syndrome.

Most of the genetic material loss in del2q37 patients is de novo [2, 9] and therefore, their parents are healthy. However, in some cases, polymorphisms of the 2qter region are found in one of the healthy parents that may predispose to important deletion in the 2q37 location in the offspring [10]. Given the pregnancy history of the patient’s mother and the macrocephaly also present in the father, further genetic testing (e.g., genome or exome sequencing) in the patient and their parents could give important information for genetic counseling and determining additional genetic changes in the patient that may be associated with the symptoms that she presents.

The deletion in the terminal region of one of the chromosome 2 in our patient led to the elimination of 60 protein-coding genes (Fig. 1) including the HDAC4 gene which is one of the most important genes driving the symptoms in del2q37 patients [6]. Furthermore, there is no clear association with length of deletion (as previously hypothesized) and clinical phenotype; even more, larger deletions (9.5 and 10.1 Mb) have been associated with higher IQ whereas smaller deletion with lower IQ (6.6 and 7.9 Mb) [11]. Deleted genes in our patients also include genes that, in homozygosis, have been associated with different syndromes and pathologies (Table 1) such as Bethlem myopathy 1 (gene COL6A3), advanced sleep phase syndrome (PER2), Barber-Say syndrome (TWIST2) and susceptibility to type 2 diabetes mellitus 1 (CAPN10) as reported in OMIM database. However, due to the lack of information about which of these genes’ monosomy are tolerated and which are not, no conclusions can be drawn regarding their role in the phenotype.

Early diagnosis of del2q37 syndrome is important because it provides information for appropriate surveillance, interventions and genetic counseling. Moreover, social and language skills can improve greatly with age, as seen within a short time in our patient, making the syndrome more difficult to diagnose in later years [12]. Given the broad range of clinical manifestations that del2q37 patients can present, a timely diagnosis of this disease, as in the presented case, will allow monitoring some of the most common as well as unforeseen complications. For instance, some del2q37 patients were reported to have antibody deficiencies, leading to lower respiratory infection [6]. Therefore, our patient requires assessment and monitoring by different medical specialties.

In Colombia, few del2q37 patients have been reported with important phenotypical differences from our patient. One was an 8-year-old female with brachydactyly type E, autism, developmental delay (including verbal communication), common dysmorphic features and mild tricuspid valve regurgitation [13]. The other was an 18-year-old female with congenital heart malformations, developmental delay, autism, arachnodactyly and a concurrent pathogenic duplication in the 2p25.3p24.3 region [14].

In conclusion, we report a patient with a deletion of 5.9 Mb in the subtelomeric or distal region of the long arm of chromosome 2 and clinical manifestations compatible with del2q37 syndrome. Interestingly, this patient, up to her age at the time of this case report (3 years old), had a mild phenotype with only delay in verbal communication, umbilical hernia, hypotonia, facial dysmorphic features and an unusual feature, macrocephaly. Given the timely diagnosis, surveillance and management of complications can be addressed properly. This case provides further information for expanding the phenotypic characterization of del2q37 syndrome.

## Data Availability

The datasets used and/or analyzed during the current study are available from the corresponding author on reasonable request.

## Abbreviations

array-CGH: Microarray-based comparative genomic hybridization
del2q37: Deletion in the 2q37 cytoband
HDAC4: Histone Deacetylase 4
NCBI: National center for biotechnology information
Mb: Megabases
